# Longitudinal Radiographic Bone Density Measurement in Revision Hip Arthroplasty and Its Correlation with Clinical Outcome

**DOI:** 10.3390/jcm12082795

**Published:** 2023-04-10

**Authors:** Philip P. Roessler, Jakob Eich, Dieter C. Wirtz, Frank A. Schildberg

**Affiliations:** 1 Department of Orthopedics and Trauma Surgery, University Hospital Bonn, 53127 Bonn, Germany; 2 Gelenkzentrum Mittelrhein, 56068 Koblenz, Germany

**Keywords:** bone density, software, grayscale, hip arthroplasty, clinical outcome, Harris hip score

## Abstract

The subjective analysis of conventional radiography represents the principal method for bone diagnostics in endoprosthetics. Alternative objective quantitative methods are described but not commonly used. Therefore, semi-quantitative methods are tested using digital computation and artificial intelligence to standardize, simplify, and ultimately improve the assessment. This study aimed to evaluate the correlation between relative density progressions and clinical outcomes. Radiographs and clinical examinations before and 24 and 48 weeks after surgery were obtained from sixty-eight patients with a modular hip stem. For the calculation of the relative bone density, the modal gray values of the Gruen zones were measured using ImageJ and were normalized by gray values of the highest and lowest ROI. The clinical outcomes were measured according to the Harris hip score before evaluating them for correlations. Analyses were performed separately for subgroups and bone regions. The Harris hip score increased from 44.15 ± 15.00 pre-operatively to 66.20 ± 13.87 at the latest follow-up. The relative bone density adjustment of Gruen zone 7 showed a significant correlation to its clinical outcome. Other bone adaptations could be realistically reproduced and differences by regional zones and patients’ histories visualized. Next to the simplicity and that no additional examination is required, the method provides good semi-quantitative results and visualizes adaptations, which make it suitable for use.

## 1. Introduction

The periprosthetic bone remodeling following total hip arthroplasty (THA) is monitored using conventional radiography (CR), which is prone to bias due to a certain inter-rater variability. Other means of measuring, including dual-energy x-ray absorptiometry (DXA), volumetric computed tomography (qCT), or dual-energy CT (DECT) have been described in the literature, but they are not used broadly because of high costs and impracticability [1,2,3]. However, quantitative support is particularly useful in revision arthroplasty with complex histories and challenging bone conditions, for which there are far fewer experience reports and data than for courses after primary prostheses. A quantitative parameter helps to assess the situation and to make and legitimate a decision and what has been missing so far, especially in revisions. Recent studies have hence reported a new method to quantify relative radiographic bone density with an easy-to-use algorithm on plain radiographs with modular revision stems [4]. The reported method involves a semi-quantitative measurement of the relative periprosthetic bone density using an open-source image processor (ImageJ) to evaluate sequential anterior-posterior (a.p.) CR images of the operated hips. Individual bony characteristics such as a thin cortical frame, which might lead to significant differences in DXA t-scores, are avoided due to a thorough check by the user [5]. Metal artifacts, which may interfere in DXA or qCT measurements following THA revision, are also not an issue due to manual processing within the method proposed by Roessler et al. [4,6,7]. Moreover, the measurement is cheap, does not involve any additional radiation (besides the one that is needed for CR anyway), and can be performed by every clinical specialist after a short introduction. As a definition, we need to delimit the physical bone mineral density (BMD), which is a measure of mass per volume, to the relative (radiographic) bone density, which is characterized by transmission or darkening on a roentgen film and can be expressed as a gray value. A proof-of-principle with 18 clinical case analyses over time, without the differentiation of previous patient history, suggests that the reported method is feasible and has the potential to improve clinical practice. Moreover, a correlation with objective results of 86 patients by clinical raters of CR implies that this semi-quantitative measurement yields robust and reproducible clinical results [4].

The present study was designed to further validate the tested method and bring it one step closer to clinical use as a quantifiable follow-up parameter. The aim, in addition to an additive enlarged study cohort of revision patients, was to achieve a correlation with a clinically validated outcome measure that can be determined without risk as preliminary work for a comparison to the quantitative methods used with ionizing radiation. For this purpose, the commonly used Harris hip score (HHS) was chosen as a comparative parameter. In addition, the goal was to test whether the method could differentiate various subgroup characteristics, such as different patient histories. The hypothesis established was that there is a correlation between the progressions of established follow-up scores after hip prosthesis implantation and the relative radiographic bone densities, and secondly, there are different quantitative characteristics for the chosen subgroups.

## 2. Materials and Methods

### 2.1. Patients

This retrospective single-center cohort study was based on data from 68 patients who, as in the proof of principle, were implanted cement-free with the MRP-TITAN modular titanium stem (Peter Brehm GmbH, Weisendorf, Germany), which is designed with eight longitudinal ribs in the anchoring stem component for initial, distal diaphyseal press-fit fixation. The stem is used primarily for advanced metaphase bone defects that extend into the diaphysis and require fixation and force transmission in the deep diaphysis and isthmus of the femur, which is why it is used primarily in revisions but also in occasional cases as a primary stem. The data from the patient management database were anonymized and analyzed on an aggregated basis. Inclusion criteria were the above-mentioned implanted uncemented femoral component, available standardized radiographs in two projections, as well as concurrent orthopedic clinical follow-ups at a minimum of three different time points between 2010 and 2019. The specific times comprise one perioperative (clinical assessment just prior to surgery and radiological follow-up within three days after surgery) and two postoperative follow-ups at 24 and 48 weeks. All reasons for the stem insertion were included. The study did not define any exclusion criteria. To obtain a more differentiated analysis, we divided the cohort into four categories similar to the German Arthroplasty Registry according to the respective reason for the implantation of the MRP stem: “septic loosening” with two-stage revisions and a Girdlestone situation in between, “aseptic loosening”, “primary stem implantation” in large bone defect situations where standard primary stems were not sufficient, and “others”, with cases in which the cause of revision of the previous stem was neither septic nor loosening.

### 2.2. Clinical Evaluation

For clinical evaluation, which is crucial for the targeted comparison between clinical outcomes and relative bone density changes here, all patients were assessed preoperatively as well as at regular intervals postoperatively. The Harris hip score, as a validated tool to measure the functional status in hip pathologies, was selected as the primary clinical outcome parameter to standardize the measurement of the respective states. Additionally, the reason for revision, the femoral bone defect, and the implanted stem size were determined and documented. Postoperative dislocation and potential complications such as major orthopedic surgeries or pathologies in other joints were documented along the way. The routine follow-up examinations were again measured according to the HHS. The preoperative clinical scores were used as a baseline to calculate the difference over time (ΔHHS). This allowed us to analyze either the improvement or deterioration of function and pain at 24 and 48 weeks after revision.

### 2.3. Objective Radiologic Evaluation

**Figure 1:**
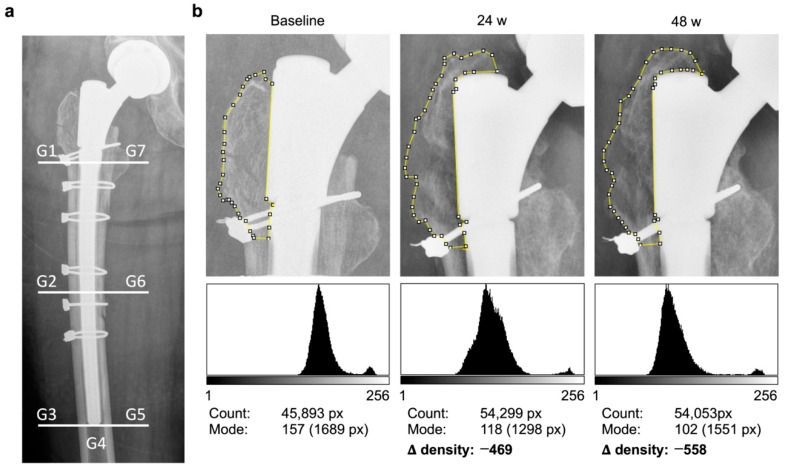
Workflow of the semi-quantitative relative bone density measurement method using ImageJ involving (**a**) the subdivision into seven periprosthetic Gruen zones (G). (**b**) Example case of proven bone density loss in zone G1 after prosthesis revision due to periprosthetic fracture. After subdividing the proximal femur into the periprosthetic zones on a conventional AP pelvis standing radiograph, each region was individually tagged with the polygon tool as depicted for G1. ImageJ defined the total number of enclosed pixels (Count) and the measured modal gray values (Mode) together with the corresponding number of pixels as in the displayed histograms. Due to the modal value, external influences, such as the cerclage in this case, had no influence on the result. Then, the modal gray value was normalized with the results of the measurement of the high and low zones of each radiograph. The differences of these scores to the perioperative baseline objectively represented the remodeling of bone density as Δ-density and could thereby prove the decrease of bone density in G1 in the present case.

Within three days after implantation, as well as during each clinical follow-up visit, plain radiographs (AP pelvis standing and involved hip axial according to Lauenstein) were taken. The evaluations were conducted in AP pelvis standing radiographs using IMPAX EE (Agfa HealthCare GmbH, Bonn, Germany) and ImageJ 2.0 (Wayne Ras-band, National Institute of Health, Bethesda, MD, USA) and were performed as described in detail in the proof-of-principle study [4] (Figure 1).

In brief, radiographs were taken in standardized settings and in accurate positioning confirmed by a senior radiologist. The 3032 × 2520 pixel (px) images were imported to ImageJ in an uncompressed 8-bit grayscale format. There, as the first step, two 50 × 50 px regions of interest (ROI) were created in areas with the lowest (lo) and highest (hi) optical density to determine low and high tone densities. The hi-ROI was manually located in the femoral ball head or proximal stem, whereas the lo-ROI had to be between the legs or at the edge without a soft tissue shadow. Hi- and lo-histograms typically showed a spike; thus, no influencing error had to be assumed. For further processing, peak values and their frequencies within the respective ROI were collected.

In the second step, the seven so-called Gruen zones (G) as defined by Gruen [8] were traced, taking care to ensure that foreign material had no influence on the mode value. In the third step, a comparable score, dependent on the contrast set and the surrounding patient characteristics, was generated from the measured values. For this purpose, the relative bone density was calculated according to the following formula:

$$\frac{\left(Mode\;Peak\;G\times Frequency\right)-\left(Peak\;lo\times Frequency\right)}{\left(Peak\;lo\times Frequency\right)/\left(Peak\;hi\times Frequency\right)}$$

This allows each measured gray value to be normalized based on the high and low gray value field displayed in the image based on the given intensity frequency. To improve the comparability and to analyze the dynamics of the relative bone density over time, determined values for 24 and 48 weeks were subtracted from baseline data to generate delta values (Δ) as a relative measure.

Following this, the Δ-density scores were compared in terms of their dynamics in the patients and then correlated to the respective clinical outcome score by comparing with ΔHHS. This was then investigated more closely concerning the different Gruen zones and the different indications for the revision stems.

### 2.4. Statistical Evaluation

The recorded, anonymized values were transferred and exported to Microsoft Excel 365 (Microsoft Corporation, Redmond, DC, USA), and all analyses were performed using Graph Pad Prism 7 (Graph Pad Inc., La Jolla, CA, USA). Two-tailed Student’s t-tests were used to compare differences between groups. Linear regression with a 95% confidence interval was performed to correlate objective and subjective results, followed by the calculation of two-tailed Pearson’s correlation coefficients with the same confidence interval. Data are given as means ± standard deviation (SD), as are ranges if not indicated otherwise. The level of significance was set at *p* < 0.05.

### 2.5. Ethical Approval

The study was conducted in accordance with the Declaration of Helsinki and was approved by the Institutional Ethics Committee of the University of Bonn (protocol code 224/17 and 8 August 2017).

## 3. Results

### 3.1. Patients

Out of more than 500 patients who underwent surgery at our department between 2010 and 2019, according to the inclusion criteria, 68 were selected. Table 1 shows the demographic patient characteristics (Table 1). The gender and side of operation showed equal distribution. The main cause of the revisions was the aseptic loosening of the previous prosthesis (36.75%), followed by septic reasons of removal (27.94%).

The most commonly analyzed perioperative radiographic femur defects were categorized as 3a, 3b, and 3c (61.8%). Due to the open inclusion criteria, the used stem sizes represented a wide range between 13 × 140 mm and 25 × 200 mm. During the study period, no patient lost his stem; thus, no further patient had to be excluded based on a primary endpoint. Moreover, none of the patients had major orthopedic surgery in the period under consideration up to 48 weeks after surgery. In seven cases (10.29%), a single or repeated dislocation event occurred postoperatively.

### 3.2. Clinical Outcome

The functional outcome was objectively measured by HHS to be able to compare the improvement over time. As shown in Table 2, the mean preoperative score was 44.15 (±15.00). It improved to an average of 62.16 (±12.09) after 24 weeks and an average of 66.20 (±13.87) after 48 weeks. Therewith, the average difference between the control appointments preoperatively and 24 weeks postoperatively was an improvement of 18 HHS score points. By 48 weeks, this average difference increased by another 4 score points.

Additionally, on both control time points, less than 20% had a negative difference. This means that the majority benefited clinically from the intervention and mostly the largest difference occurred before the first follow-up time point. Comparatively, between the time points of 24 and 48 weeks, just small average adaptions in the positive direction could be observed.

**Figure 2:**
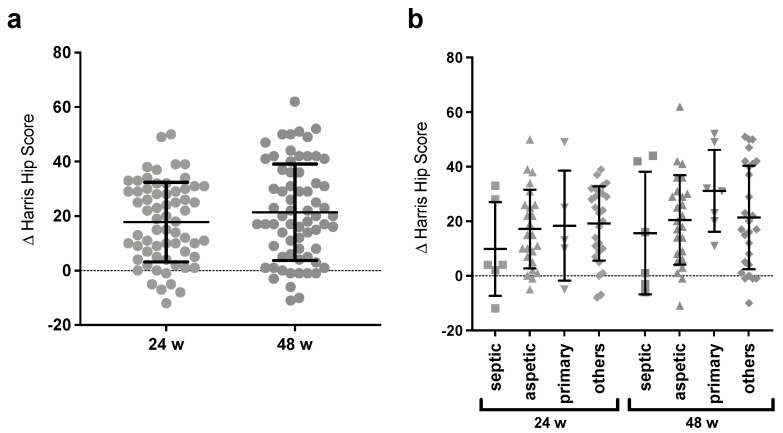
The clinical outcome after 24 and 48 weeks with (**a**) the ΔHHS development of all patients; (**b**) differences for subgroups. Clinical improvement could be seen for >85% of patients. The greatest improvements were up to 24 weeks and afterward; mainly, little adjustments and a widening of spread took place. Furthermore, a clinical improvement occurred in all subgroups, after 48 weeks the largest for the primary implantation patients, and the smallest for patients after septic loosening. No significant differences were seen between the groups.

Looking at the individual results in addition to the average (Figure 2a), it showed that, in the period of 24 to 48 weeks, the dispersion widened mainly in the positive direction while the trend remained the same, and as an effect, the upper quartile improved more noticeably than the others.

When the cases were divided according to their reasons for implantation (Figure 2b), considerable differences were found, not only in the overall clinical success but also in the time to which substantial changes occurred. Across all subcategories, there was a clear positive trend in HHS but no significant differences were seen between the groups. However, patients with prior septic loosening experienced the least improvement at both 24 and 48 weeks. After 24 weeks, the group “others” with mainly mechanical reasons for revision benefited the most. Nevertheless, after 48 weeks, patients with primary stem implantation showed the greatest clinical improvement compared to preoperative controls and a further major increase in the mean between weeks 24 and 48, which was not seen in the other subgroups.

### 3.3. Radiographic Outcome

**Figure 3:**
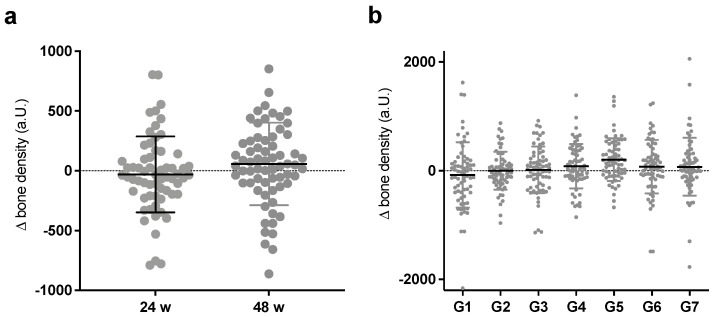
(**a**) Average Δ-density after 24 and 48 weeks; (**b**) averages G1 to G7 after 48 weeks. There was non-significant mean density deterioration after 24 weeks and improvement after 48 weeks with increasing outliers over time, as some stems showed progressive loosening or stress shielding, while others showed good bone remodeling and solid ingrowth. Thereby, clear differences could be seen for the Gruen zones with the strongest outliers and widest spreads in both directions in proximal areas and the least changes in distal areas.

The first step toward the aim of the study was to apply the presented method of relative bone density to a larger collective and to test its validity and usability. For this, the differences in the relative bone density scores of the full cohort between the time points perioperatively and 24 and 48 weeks were measured and calculated (Figure 3a).

As shown in Figure 3a, no significant difference between the time points was detectable, although a tendency towards an increasing mean was visible. As expected with the open inclusion criteria, there was a wide distribution of scores after 24 weeks, which extended to 48 weeks. Furthermore, again, a major increase took place in the first 24 weeks after surgery, and only minor adjustments followed. A more specific analysis along the periprosthetic Gruen zones showed the widest spread for the proximal zones G1 and G7 and the smallest spread for the distal zone G4 after implantation of the distally fixed MRP-TITAN, but again without significance (Figure 3b). Moreover, there was no clear direction, but both the strongest density increase and decrease were found in G1 and G7 in equal measure.

**Figure 4:**
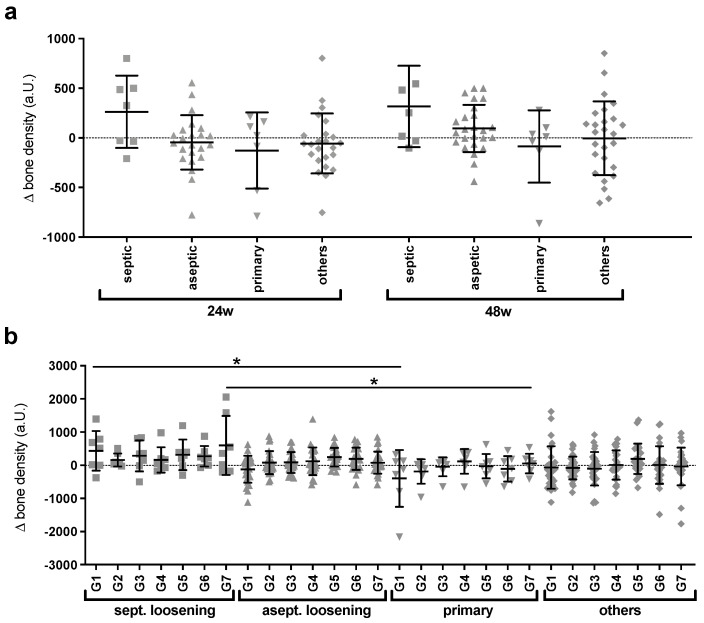
(**a**): Average Δ-density of subgroups after 24 and 48 weeks; (**b**) average G1 to G7 of subgroups after 48 weeks. Different quantitative adjustment characteristics emerged for the four subgroups, and the method detected significant differences (* *p* < 0.05) between the two subgroups septic loosening and primary for both zones G1 and G7. There was, in total, a large increase in bone density after septic causes, in contrast to a small decrease after primary stem implantation, and no consistent trend for the subgroups aseptic loosening and others. Even subdivided into the Gruen zones, there was no uniform adaptation behavior, and the cases were very individual. Nevertheless, it can be observed again that the largest variations were observed in the two proximal zones.

The next step in achieving the goal of the study was to divide the group by its subgroups and see if the method could detect and differentiate among various adaptive characteristics. Thereby, interesting progression differences were observed (Figure 4a), as previously observed with the Harris hip score.

In patients with a preoperative history of septic loosening, there was a trend toward bone density gain at 24 weeks that remained at 48 weeks. In contrast, the primary implanted stems showed an overall tendency toward bone loss. Further subdivided according to the Gruen zones, this differentiation between the two subgroups by the method was shown to be significant (*p* < 0.05) for the two proximal zones G1 and G7 (Figure 4b). In contrast, in the other two subgroups, there were no remarkable or significant average changes at both 24 and 48 weeks, but there were large differences in dispersion and individual progressions.

### 3.4. Correlation of Clinical and Radiology Aspects

**Figure 5:**
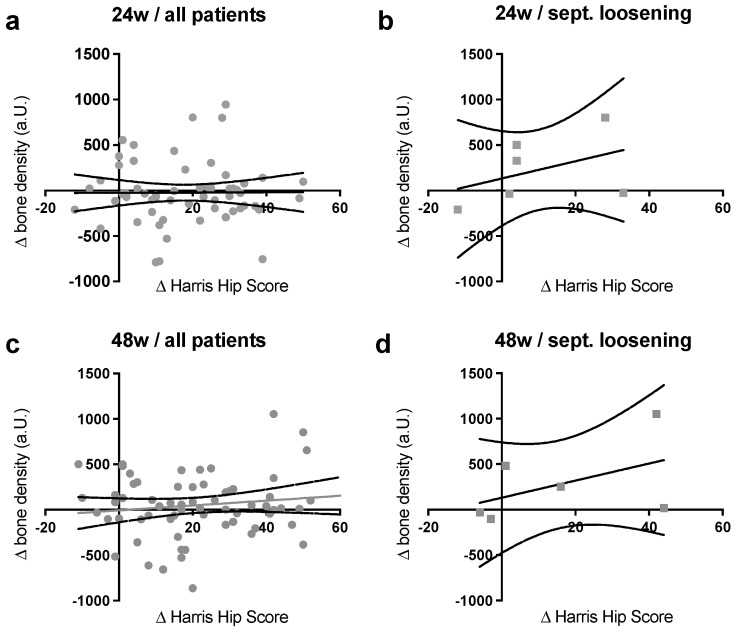
Relation of the average clinical and radiographic developments (**a**) of all patients after 24 weeks; (**b**) septic loosening after 24 weeks; (**c**) all patients after 48 weeks; (**d**) septic loosening after 48 weeks. Linear regressions after 24 weeks of the entire cohort depicted no correlation tendency, a visible increase after 48 weeks (but also no statistical significance), as well as a decrease in outliers. The subgroup of septic loosening, with the most significant bone change, showed a strong positive connection between the average bone density and the clinical development after 24 and 48 weeks, but, here, no significance could be proven.

In the next crucial step, the Δ-density values after 24 and 48 weeks, measured and calculated with the new method for the entire population, were correlated with the ΔHHS scores (Figure 5a,c).

In this first generalized part, there was no correlation tendency after 24 weeks and a positive—but not significant—correlation after 48 weeks. In a second step, a more precise examination of the subgroups that had the clearest bone adjustment after the stem implantation, the patients with septic loosening in their patient’s reports, was therefore conducted (Figure 5b,d). For this group, a clear positive correlation between the two parameters was found at both 24 and 48 weeks, which meant that an increase in bone density was accompanied by an improvement in clinical outcomes.

**Figure 6:**
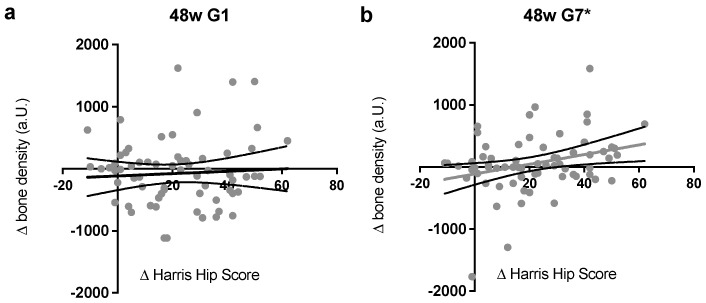
The correlation of clinical and radiographic development after 48 weeks (**a**) in G1; (**b**) in G7 (significant * *p* < 0.05). The bone density in the proximal zones G1 and G7, which showed previously the biggest adjustments after MRP-TITAN, and the difference in Harris hip scores 48 weeks after implantation showed a clear positive dependence. Especially in G7, a significant correlation (* *p* < 0.05) with a Pearson correlation coefficient of 0.302 could be found for both parameters. This suggests that, for the clinical outcome during the first year after the MRP-TITAN implantation, the development of the proximal periprosthetic bone segment is crucial.

Lastly, zones G1 and G7 were analyzed in more detail for potential correlations (Figure 6), as the largest differences were measured in the proximal zones. Here, the most convincing correlation was observed. For zone G1 (Figure 6a), a positive correlation can be assumed, but in the present study, it could not with significant evidence proven.

For G7 (Figure 6b), a significant positive correlation (*p* < 0.05) between the changes of the relative bone density and the Harris hip score after 48 weeks was detected; therefore, a strong relation between the semi-quantitative method and the clinical outcome can be considered.

## 4. Discussion

The most important achievement of the presented study is the demonstration that relative bone density measurement can accurately differentiate between different patterns of bone changes and that at least one of the Gruen zones can reflect clinical outcome parameters as a progression parameter, thus providing a significant advancement beyond previous research. Additionally, our study highlights the practicality and accessibility of this method for clinicians by utilizing standard radiographs, without requiring complex imaging techniques or procedures, and reconfirms the previously seen promising study results of the method as reliable and reproducible on another significantly enlarged cohort. Within this study, it was possible to generate quantitative statements for the periprosthetic bone using the new method, which was described and tested by Roessler et al., 2019 [4] with the first proof of principle. Furthermore, a positive correlation between bone remodeling in G7 and the clinical outcome of the patients one year after stem implantation measured with the Harris hip score could be noted. Therefore, our hypothesis that there is a correlation between the progressions of the relative radiographic bone density developments and an established follow-up score could be accepted only for the proximal region in the case of Gruen zone 7.

More precisely, it was not possible to find a direct significant correlation between the average relative bone density value and the clinical outcome without division into femoral regions. Whereas, with a division into Gruen zones, the medial trochanteric region G7 correlated significantly (*p* < 0.05) with the clinical outcome. In addition, it was possible to demonstrate different patterns of bone changes in subgroups of patients with varying indications for implantation, and for at least two of these subgroups, our method allowed for the quantitative identification of significantly different adaptation characteristics. This highlights the potential of our approach to provide valuable insights into the bone-implant interface and to guide treatment decisions in a more personalized manner.

In the present study, the studied patient cohort was similar in distribution to the values in the German prosthesis registry [9] for sex, age, body mass index, and reasons for stem implantation and thus seems suited to make more global assumptions. The clinical outcome shown here is also comparable to studies in which medium- and long-term analyses were performed with the MRP-TITAN [10]. The relatively short interval of the study at 24 and 48 weeks postoperatively reflects the main period of change of both bone [11,12,13,14] and HHS [15] but implies that statements with a more long-term reference must be interpreted with care.

The hypothesis that the relative bone density correlates with improved clinical outcomes was only significantly demonstrated for G7. The results for the other Gruen zones, G1 to G6, were not as representative, as although there appeared to be slight positive associations, no significant correlation could be proven. This is in line with the result of [16] that metaphyseal bone augmentation not only improves proximal stability but also the functional outcome of the patient. This suggests that the HHS can be assumed to be a comparable progression parameter to bone density, especially in the proximal sections, and that the other sections do not correlate as strongly with clinical outcomes. Consequently, the lack of correlation in the distal regions may not be interpreted as a limitation of the tested method.

To prove the validity of the grayscale values, it is necessary to compare the results of existing studies after MRP-TITAN stem implantations. The most-used method for this is currently the DEXA scan, which on the one hand, can detect already small bone changes [17], but on the other hand, it also requires additional imaging only for bone densitometry. Similar to these published studies [14], we measured a minimal change in distal sections and the strongest deviations in proximal sections. We were also able to demonstrate expected courses of bone density depending on the cause of implantation. In septic loosening, there is often a two-part operation with a Girdlestone situation in between, without loading of the bone, in which bone density is lost. After implantation, this density is regenerated by loading the joint again, and an increase can be observed. In contrast, primary implantation replaces physiological load transfer, more weight is transferred through the prosthesis, and thus the bone is unloaded, resulting in a decrease in bone density.

It remains to be considered that, with this method a two-dimensional image is examined and, therefore, there are some limitations to the score. For instance, identical positioning and focus settings are necessary and, due to possible overlays, changes in the surrounding soft tissues and possible shifts of the zones due to periprosthetic bone adaptions affect the result [6,18,19]. However, no further examination is required for this measurement and, therefore, no further radiation exposure and no additional financial expenditure. For the quantitative validity analysis of this method, a further study directly comparing the measured results of relative bone density and the DEXA score and an extension to further implants such as standard primary stems would be recommended. In addition, further large-scale studies should investigate whether the variability of the patient cohort, different etiologies, and body mass index might affect the accuracy of the results and how the current method could be further developed.

After previous studies, the conventional radiographic analysis failed to detect some significant bone changes in ROI compared to the DEXA method [20], and only considerable changes in the bone can be imaged [21]. However, in the clinical setting for diagnosis, the changes compared to previous images play a greater factor [22]. Therefore, an improved methodology of the objective analysis of radiographs still seems useful. In this regard, the method presented here is useful because it can be performed on both existing and newly acquired images and provides an objective score that can be used as a progression parameter according to our results. A further major advantage is that all measurements and calculation steps are clearly defined. In our digital world, this is the basis for coding this procedure, automating it with the help of machine learning and implementing it in an X-ray analysis program. This saves additional examinations and the currently required analysis time by a human operator. Therefore, the present study was intended to serve as an intermediate step towards the automation of this method, providing the groundwork for future automated processes and showcasing the potential of digitization in orthopedics. Even if this procedure would then generate less accurate determinations than the gold standard procedure, it would still be worthwhile to be able to make a better pre-selection. This would save burdens for the patient and the social system and avoid human errors.

## Figures and Tables

**Table 1:** Patient demographics.

Item	Value
Age (y)	67.0 ± 11.0 (range 37.0–84.0)
Body mass index (kg/m^2^)	29.3 ± 6.6 (range 18.0–53.1)
Sex (no./%)	
Male	32/47.05%
Female	36/52.95%
Side (no./%)	
Right	38/55.89%
Left	30/44.11%
Indication for revision stem (no./%)	
Aseptic stem loosening	25/36.75%
Septic stem loosening	19/27.94%
Others “Implant-associated”	16/23.53%
Primary stem implantation	08/11.76%

**Table 2:** Clinical outcome with HHS preoperatively, at 24 weeks and 48 weeks.

	HHS Preoperative	HHS 24 Weeks	HHS 48 Weeks
Total	44.15 ± 15.00	62.16 ± 12.09	66.20 ± 13.87
Aseptic stem loosening	45.00 ± 12.27	61.48 ± 11.59	65.42 ± 14.60
Septic stem loosening	42.46 ± 14.36	58.23 ± 12.36	63.23 ± 11.26
Primary stem implantation	42.00 ± 12.96	57.00 ± 14.09	73.15 ± 12.24
Others	45.19 ± 12.62	67.94 ± 10.71	66.75 ± 15.38

## Data Availability

Not applicable.
